# Supplementary figures and images for: Balancing selection at a premature stop mutation in the myostatin gene underlies a recessive leg weakness syndrome in pigs
Source: PLoS Genet. 2019 Jan 30;15(1):e1007759. doi: 10.1371/journal.pgen.1007759 (PMC6370237; doi:10.1371/journal.pgen.1007759)

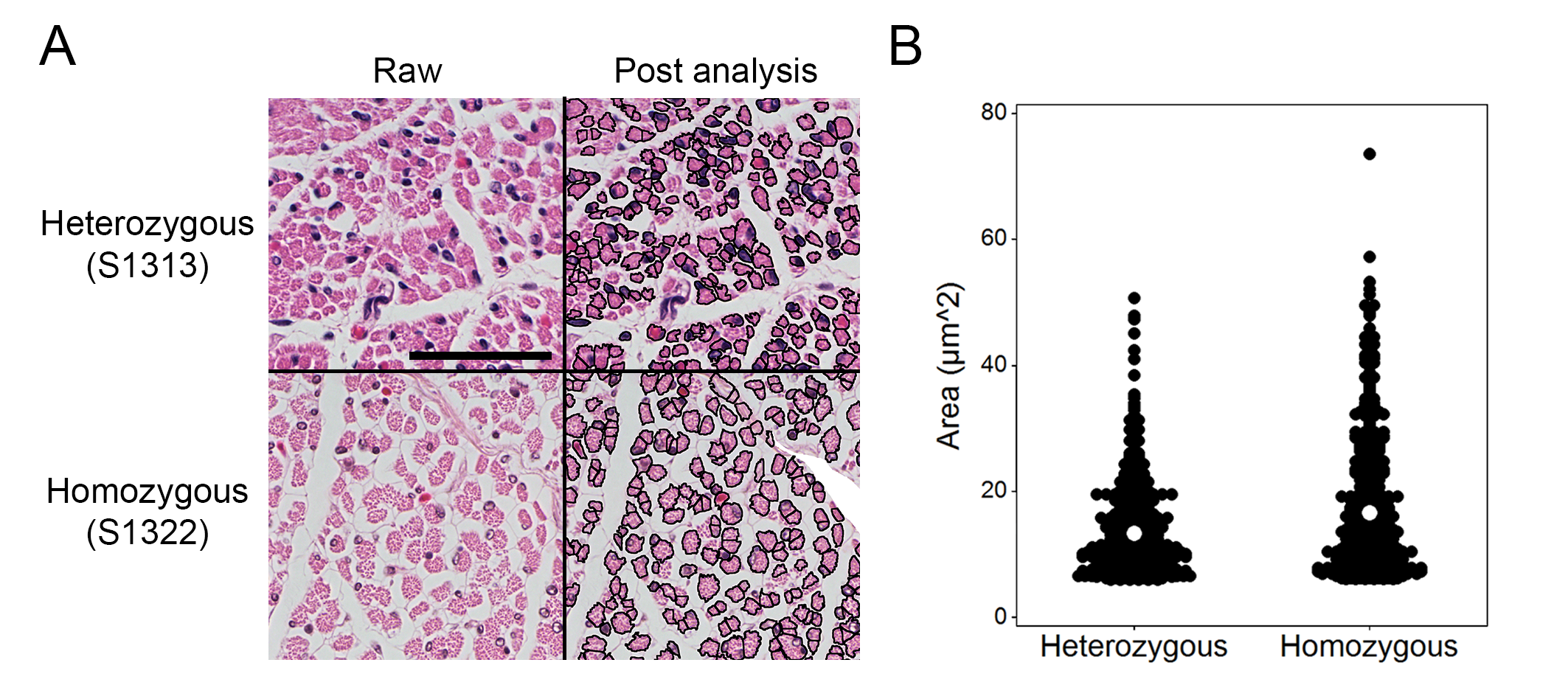

Supplement: S1 Fig — Panel A illustrates the histological presentation (raw), featuring histologically normal myofibres for both genotypes, and the results of the morphometric measurement on the same area of the sections (post analysis). Panel B, contains a dotplot chart of the comparative measurements for each myofibre taken from an identical area for each animal. The white dot indicates the median value for each group. The homozygous myofibres were significantly larger that the heterozygous (P<0.001), with a median of 13.4μm2 and 11.5μm2 respectively. This is suggestive of significant hypertrophy of myofibres in the homozygous group. Conversely, the density of myofibres is comparable in both groups (i.e. 2.2myofibres/100μm2 for both groups), suggesting no hyperplasia is present in the homozygous group. Stain: Haematoxylin and Eosin, scale bar = 50μm (applies to all figures). (TIF) [file pgen.1007759.s001.tif]
