# Supplementary material for: Balancing selection at a premature stop mutation in the myostatin gene underlies a recessive leg weakness syndrome in pigs
Source: PLoS Genet. 2019 Jan 30;15(1):e1007759. doi: 10.1371/journal.pgen.1007759 (PMC6370237; doi:10.1371/journal.pgen.1007759)
Supplement: S1 Table — All models have litter variance (σ2v) fitted. (DOCX) [file pgen.1007759.s002.docx]

| Model^¥^ | Animal Models | | Sire and Dam Models | |
| --- | --- | --- | --- | --- |
|  | σ^2^_w_ >0 | σ^2^_w_ =0 | σ^2^_w_ >0 | σ^2^_w_ =0 |
| σ^2^_u_ | 0.0072 (0.0016) | 0.0113 (0.0017) |  |  |
| σ^2^_s_ |  |  | 0.0018 (0.0005) | 0.0018 (0.0005) |
| σ^2^_d_ |  |  | 0.0016 (0.0006) | 0.0033 (0.0006) |
| σ^2^_v_ | 0.0064 (0.0005) | 0.0071 (0.0005) | 0.0063 (0.0005) | 0.0069 (0.0005) |
| σ^2^_w_ | 0.0017 (0.0005) |  | 0.0019 (0.0006) |  |
| σ^2^_p_ | 0.0594 (0.0009) | 0.0605 (0.0010) | 0.0592 (0.0009) | 0.0596 (0.0009) |
| h^2^_u_ | 0.12 (0.03) | 0.19 (0.03) | 0.11 (0.03) | 0.17 (0.02) |
| σ^2^_v_/σ^2^_p_ | 0.11 (0.01) | 0.12 (0.01) | 0.11 (0.01) | 0.12 (0.01) |
| σ^2^_w_/σ^2^_p_ | 0.03 (0.01) |  | 0.03 (0.01) |  |
| h^2^_L_ liability scale | 0.46 | 0.72 | 0.44 | 0.65 |
| LogL | 8215.64 | 8201.21 | 8257.89 | 8241.54 |

¥ - σ^2^_u,_ σ^2^_v_ and σ^2^_w_ are variances due to the additive genetic effects, litter and maternal environment effects. σ^2^_p_ is phenotypic variance. h^2^_u_ is estimated heritability on observed scale, and σ^2^_v_/σ^2^_p_  and σ^2^_w_/σ^2^_p_ are the proportion of phenotypic variance explained by the litter and maternal environment effects respectively_._ h^2^_L_ is the heritability transformed to an underlying scale, and LogL is log likelihood.
