# Supplementary material for: Balancing selection at a premature stop mutation in the myostatin gene underlies a recessive leg weakness syndrome in pigs
Source: PLoS Genet. 2019 Jan 30;15(1):e1007759. doi: 10.1371/journal.pgen.1007759 (PMC6370237; doi:10.1371/journal.pgen.1007759)
Supplement: S2 Table — (DOCX) [file pgen.1007759.s003.docx]

| Genotype | Period of measurement | | |
| --- | --- | --- | --- |
|  | Birth | up to 40Kg | At 110Kg |
| GG | 293 | 259 | 169 |
| TG | 162 | 126 | 90 |
| TT | 25 | 1 | 0 |
| Total | 480 | 386 | 259 |
| p | 0.78 | 0.83 | 0.83 |
| q | 0.22 | 0.17 | 0.17 |
| χ^2^ | 0.18 | 12.51 | 11.45 |
| α | 0.0193 | -0.1800 | -0.2103 |
